# Crystallographic Fragment Screening of the Dengue Virus Polymerase Reveals Multiple Binding Sites for the Development of Non-nucleoside Antiflavivirals

**DOI:** 10.1021/acs.jmedchem.5c01014

**Published:** 2025-09-02

**Authors:** Manisha Saini, Jasmin C. Aschenbrenner, Francesc Xavier Ruiz, Ashima Chopra, Anu V. Chandran, Peter G. Marples, Blake H. Balcomb, Daren Fearon, Frank von Delft, Eddy Arnold

**Affiliations:** † Center for Advanced Biotechnology and Medicine, 14863 Rutgers, the State University of New Jersey, Piscataway, New Jersey 08854, United States; ‡ Department of Chemistry and Chemical Biology, Rutgers, the State University of New Jersey, Piscataway, New Jersey 08854, United States; § 120796 Diamond Light Source, Harwell Science and Innovation Campus, Fermi Avenue, Didcot OX11 0DE, U.K.; ∥ Research Complex at Harwell, Harwell Science and Innovation Campus, Fermi Avenue, Didcot OX11 0FA, U.K.; ⊥ Centre for Medicines Discovery, Nuffield Department of Medicine Research Building, Old Road Campus, Headington, Oxford OX3 7FZ, U.K.

## Abstract

Dengue viruses (DENVs) infect approximately 400 million people each year, and currently, there are no effective therapeutics available. To explore potential starting points for antiviral drug development, we conducted a large-scale crystallographic fragment screen targeting the RNA-dependent RNA polymerase (RdRp) domain of the nonstructural protein 5 (NS5) from DENV serotype 2. Our screening, which involved 1108 fragments, identified 60 hit compounds across various known binding sites, including the active site, N pocket, and RNA tunnel. Additionally, we discovered a novel binding site and a fragment-binding hot spot in thumb site II. These structural findings open amenable avenues for developing non-nucleoside inhibitors and offer valuable insights for future structure-based drug design aimed at DENV and other flaviviral RdRps.

## Graphical abstract

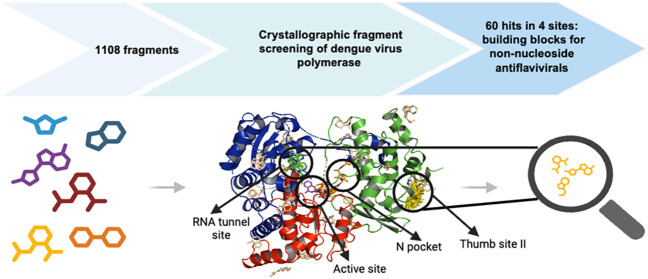

## Introduction

Flaviviruses are single-stranded RNA viruses primarily transmitted by arthropods, causing severe illnesses in humans.^1^ Over the past seven decades, the global spread and epidemic transmission of flaviviruses have been significant.^2^ The mosquito-borne dengue viruses (DENVs) infect approximately 400 million individuals annually, with over a quarter of the world’s population residing in endemic regions.^1,3^ The disease symptoms range from self-limited dengue fever to severe dengue syndrome. The number of infections has steadily increased over the last 70 years, making DENV the most widespread arthropod-borne viral disease globally.^3,4^ Strikingly, the World Health Organization has reported that around half of the world’s population is at risk of contracting dengue.^5^ Any of the four DENV serotypes (DENV1–4) causes an acute febrile disease named dengue fever. However, many infected people, including a large proportion of children, develop life-threatening forms of the disease known as dengue hemorrhagic fever and dengue shock syndrome.^6^ Prevention and control measures thus focus on vector management, as there is currently no specific treatment for dengue or severe dengue. Early detection and access to appropriate medical care are crucial in reducing fatality rates associated with severe dengue.^1,7^ Currently, Q-denga (TAK-003) and Dengvaxia are the only approved vaccines for dengue, both based on live-attenuated viruses but differing in their composition and target populations. Overall, their main drawbacks are that they do not offer uniform immunity across all four DENV serotypes and have limited applicability across different population groups.^8,9^

The DENV viral particle consists of a positive RNA strand of 11 kb that forms the viral genome, including 5′ and 3′ untranslated regions (UTR) and a 5′ cap.^10^ Upon infection, the RNA is translated into a single polypeptide chain within the endoplasmic reticulum (ER) membranes, which is then processed into ten proteins through proteolytic maturation by both viral and host cell proteases. The structural proteins, including the envelope, precursor membrane, and capsid, encase the viral RNA. The nonstructural (NS) proteins (NS1, NS2A, NS2B, NS3, NS4A, NS4B, and NS5) are expressed within the host cell but are not incorporated into the viral particle. With the assistance of host proteins, these NS proteins reorganize the internal structure of the cell, process the polyprotein to yield the mature viral proteins, replicate the viral RNA, and help the virus evade the immune system.^11,12^

NS5 is the largest and most conserved DENV protein, with approximately 100 kDa and 70% sequence identity among the four dengue serotypes. It contains two key domains: a methyltransferase domain (MTase) at the N-terminus and RNA-dependent RNA polymerase (RdRp) at the C-terminus, connected by a 5–6 residue linker (residues 266–271) that plays a crucial role in determining NS5′s overall conformation and activity. The structure of NS5 is highly conserved across flaviviruses, suggesting the potential for designing broad-spectrum antiviral compounds targeting NS5.^13^ However, there are different relative conformations between MTase and RdRp across the known full-length flaviviral NS5 structures.^6^ The MTase domain (residues 1–265) is involved in capping the viral RNA and has guanylyl transferase, N7, and 2′-O ribose methylation activities. The RdRp domain is responsible for replicating the viral RNA, which is initiated *de novo* and facilitated by the “priming loop”. Upon transition to elongation, the priming loop retreats from the active site to allow elongation of the RNA duplex.^14^ NS5 forms a larger replicase complex alongside the NS3 helicase, which assists in both polymerization and capping.^15^ Beyond its role in viral genome replication, NS5 can also suppress the host immune interferon response, either through interaction with the signal transducer and activator of transcription 2 (STAT2) protein or by influencing RNA splicing within the host cell.^11,16^ Recent structural work has provided improved detail on the conformational selection mechanism underlying STAT2 inhibition by NS5.^17^

Given that RdRp activity is absent in host cells, the RdRp domain of NS5 is a promising antiviral target for designing specific inhibitors with minimal on-target toxicity. Despite the crucial role of NS5 RdRp in the viral replication of DENV, the development of novel direct-acting antivirals has not exceeded the preclinical stage, except for the nucleoside analogue AT-752 (Atea Pharmaceuticals), a chain terminator^6^ currently in clinical trials.^18^ Despite this encouraging development and their historical success in drug development, it is known that nucleoside analogues have experienced a high attrition rate in clinical trials due to toxicity.^19^ Regarding non-nucleoside inhibitors, through a structure-based fragment screening approach, researchers have developed allosteric inhibitors that target the so-called “N pocket” of DENV and Zika virus RdRps.^20–23^ Nevertheless, retraction of the priming loop from the active site during enzyme elongation may alter the conformation of the N pocket, which may explain the weak affinities of the lead compounds developed despite the availability of multiple crystal structures of such protein–ligand complexes.^21^

The previous fragment screening campaigns (that yielded the N pocket inhibitor type) were performed using fragment cocktails against DENV3 RdRp. Here, leveraging the state-of-the-art XChem facility and pipelines,^24^ we performed a large-scale crystallographic fragment screening campaign (1108 fragments, with single fragment soaking) targeting DENV2 RdRp. Our study probed known binding sites (the active site, the N pocket, and the RNA tunnel site) and unveiled a fragment-binding hot spot (the thumb site II), providing insights for the development of novel DENV2 non-nucleoside inhibitors. Furthermore, these findings offer a framework for identifying and targeting analogous pockets across flaviviral RdRps.

## Results

### DENV2 RdRp Crystallizes in a Ligand-free Form that Diffracts to High Resolution

The DENV2 NS5 RdRp apo crystal was solved with data diffracting to a 1.56 Å resolution (PDB 7I2X). A single molecule was observed in the asymmetric unit (ASU) in the *I*222 space group, consistent with previously reported structures.^21,25^ The structure closely aligns with the previous highest-resolution apo (PDB entry 6IZY, 2.11 Å resolution) and ligand-bound (PDB entry 5K5M, 2.01 Å resolution) DENV2 RdRp structures (RMSD = 0.385 Å and RMSD = 0.787 Å, respectively). Despite the improved resolution, the apo structure presented similarly disordered regions as the previous one (Figure S1), including a disordered priming loop (similarly to PDB 6IZY and conversely to PDB 5K5M, with a small molecule bound in the N pocket) and an ordered motif G (similarly to PDB 6IZY and conversely to PDB 5K5M). Preliminary screening of this crystal form with a small subset of fragments validated its suitability for large-scale crystallographic fragment screening.

### Crystallographic Fragment Screening Comprehensively Maps Many Binding Sites in DENV2 RdRp

We soaked 1108 fragments into the aforementioned RdRp apo crystals. Soaking of crystals with 10% dimethyl sulfoxide (DMSO) for 1–3 h showed a minor effect on diffraction quality, yielding 918 data sets with resolution between 1.56 and 2.43 Å (Figure 1A). Notably, ∼60% of these data sets diffracted to a resolution better than 2.0 Å, with an average resolution of 2.1 Å overall. After thorough refinement of the ground-state model, the Pan-Dataset Density Analysis (PanDDA)^26,27^ and PanDDA2 ^26^ pipelines were run, identifying a total of 35 hits. Additionally, given the presence of several disordered loops and relative crystal heterogeneity, we ran the Cluster4x algorithm.^28^ This tool analyzes and clusters multidata set crystallographic data, grouping similar data sets together based on reciprocal lattice intensities or C-α atom positions, thereby improving the signal-to-noise ratio. PanDDA2 was then run on these preclustered data sets, which enabled the identification of an additional 25 hits, a 73% improvement. Following meticulous modeling, data curation, and cross-reviewing, a total of 60 fragment-bound structures (with 69 binding events, with 7 fragments bound in two sites and 1 fragment in three sites) and one ground-state structure of DENV2 NS5 RdRp were validated and subsequently deposited in the Protein Data Bank (PDB). PDB identifiers, names, ligand descriptors, and data collection and refinement statistics are available in Tables S1 and S2.

**Figure 1:**
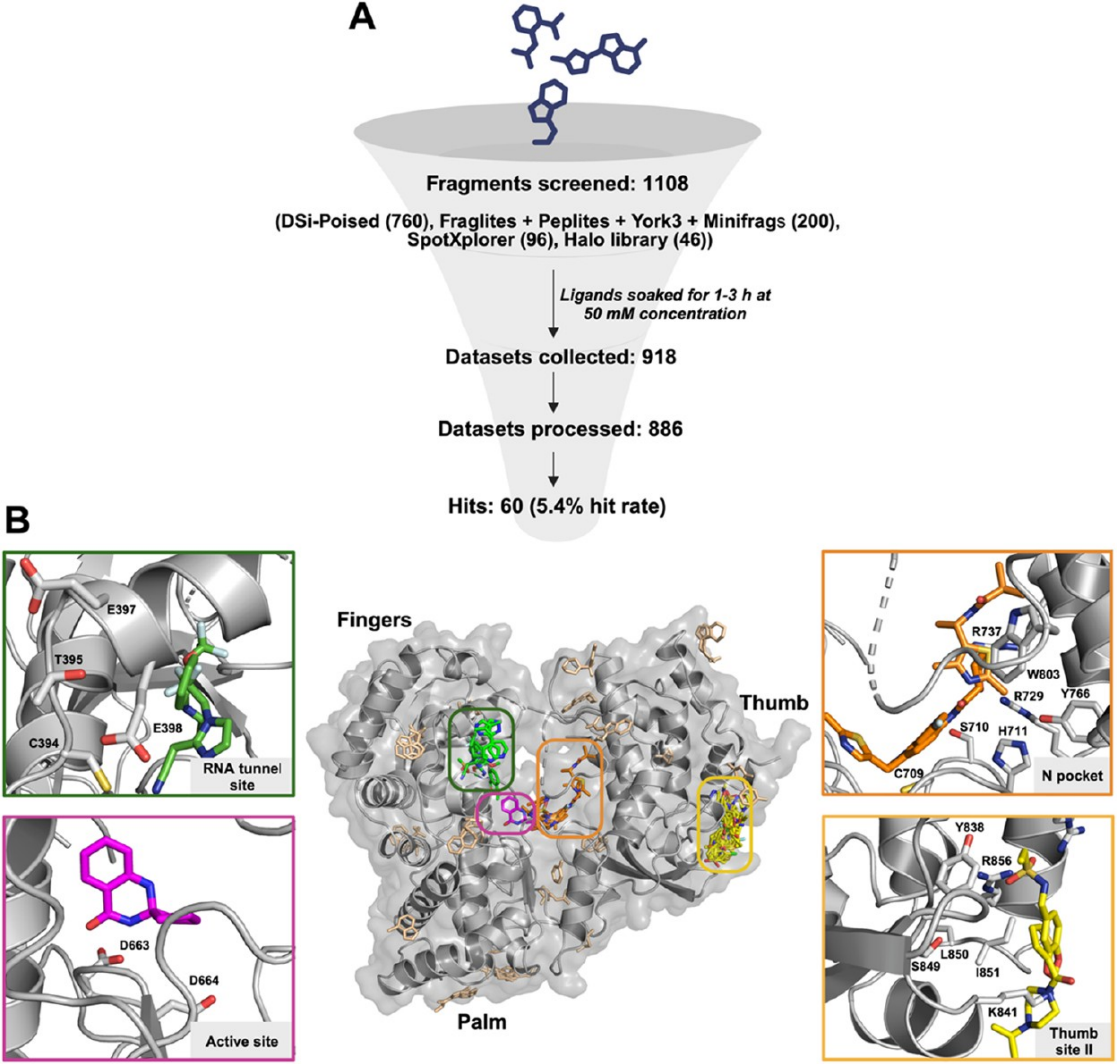
Bound fragments comprehensively sample the whole DENV2 RdRp. (A) Overview of the fragment screening campaign presented in this study. (B) Crystallographic fragment screen reveals multiple binding sites: we selected four sites that are highlighted in green, pink, orange, and yellow boxes. The green box highlights binders in the RNA tunnel site, the orange box showcases N pocket (or primer grip site) binders, the pink box indicates the single active site binder found, and the yellow box shows binders for the novel thumb site II. The remaining fragments are displayed in light brown. All of the fragment hits are depicted as sticks. The protein backbone is represented as a gray cartoon.

We identified multiple hits away from the active site, many on the surface of the protein (Figure 1B). However, it remains a challenge to rationalize and validate the putative allosteric binding pockets in the absence of fragment-originated small molecules that can be assayed in biochemical inhibition or binding assays.^29^ Based on published structures of RdRps from the *Flaviviridae* family deposited in the PDB (e.g., RdRps from flaviviruses as DENV, Zika virus; and from hepaciviruses as hepatitis C virus; HCV), we have nominated in the current study four sites with the potential of driving development of non-nucleoside small-molecule inhibitors: one active site,^15,17,30^ and three allosteric sites; N pocket (or primer grip site),^21,25,31^ RNA tunnel site,^32–34^ and thumb site II^35,36^ (Figure 1B). Figure S2 displays all hits grouped in the previous four top sites.

### Fragment Hit Binds in the DENV2 RdRp Active Site, Recapitulating the Initiating ATP

RdRps belong to the template-dependent nucleic acid polymerase superfamily. While their sequence and length are variable, they contain seven conserved motifs (from A to G) essential for nucleotide selection and catalysis. Each of the motifs adopt a specific and conserved fold and is encompassed into one of three subdomains: fingers, palm, and thumb, in analogy to the polymerase domain’s resemblance to a cupped right hand.^12^ The palm domain spans residues 497–542 and 601–705 (Figure S1), comprising a small antiparallel β-strand platform (β7 and β8) surrounded by eight helices.^1,12,31^ The domain is the most structurally conserved region among all of the known polymerases. The catalytic active site in motif C—with the strictly conserved residues GDD (G662, D663, and D664 in DENV2)—is located in the turn between strands β7 and β8 (Figure S1 ^25,37^ ).

We identified one fragment (Z48978335) in the DENV2 RdRp active site cleft (Figure 2A). The D663 backbone NH and the N610 side chain are hydrogen-bonded to the carbonyl of the fragment, while Y607 is engaged in a T-shaped π–π stacking interaction with the pyrimidine ring of Z48978335. The amide NH in the dihydroquinazolinone moiety of the fragment also displays a hydrogen bond with the oxygen atom of carboxylate D663 (Figure 2B,C). A similar positioning is observed for ATP bound to the related Japanese Encephalitis Virus (JEV) RdRp (PDB 4HDH), which might be the initiating nucleotide for the *de novo* synthesis in flaviviral RdRps.^38^ ATP forms a hydrogen bond with D668, residue that is analogous to D664 in DENV2 RdRp^39^ (Figure 2D). A superimposition of the fragment (magenta) and ATP (cyan) within the same binding pocket (Figure 2E) highlights the fragment’s binding position relative to the naturally occurring substrate.

**Figure 2:**
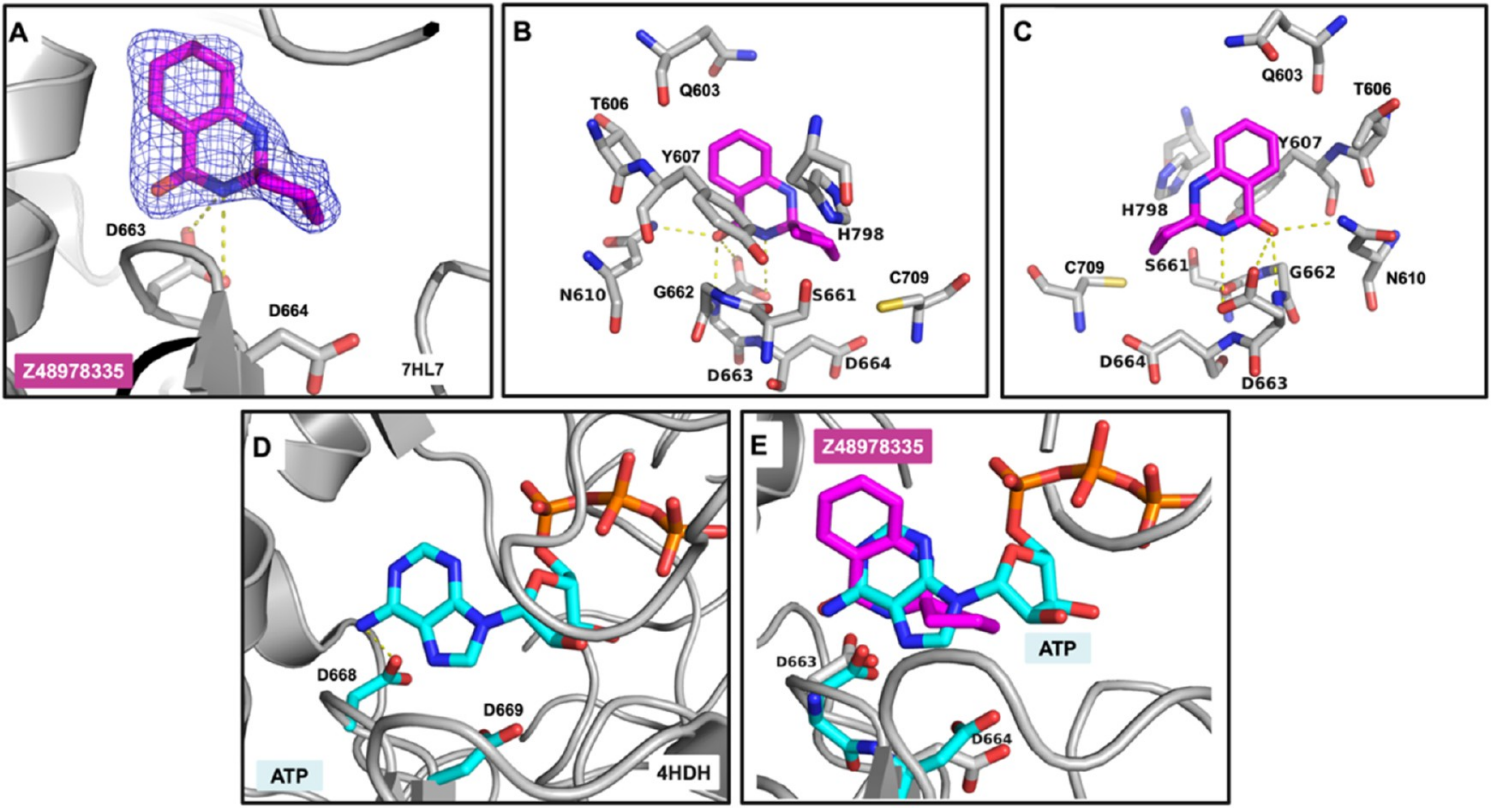
Fragment binding in the active site cleft. (A) Fragment binding in the active site cleft of DENV2 RdRp (PDB entry 7HL7). The bound fragment is shown with pink sticks surrounded by a blue mesh corresponding to the PanDDA event map at the 1σ contour. (B) Residues surrounding Z48978335 and (C) analogous to (B) with a 180° rotation in the *x*-axis. Polar interactions in panels (A–C) are displayed as yellow dashed lines. (D) Binding of ATP bound to the Japanese encephalitis virus (JEV) RdRp (PDB entry 4HDH), forming a hydrogen bond (yellow dashed line) with D668, which is analogous to D663 of DENV2 RdRp. (E) Superimposition of the bound ATP to the JEV RdRp onto the DENV2 RdRp complexed to Z48978335.

### Previously Described N Pocket Is a Hot Spot for Fragment Binding

This site was designated as the “N pocket” by Novartis scientists in a previous fragment screening campaign^22^ and is in a similar location to the Palm I in HCV NS5B.^35,36^ It is a binding cleft between the palm and the thumb subdomains, with the “exit dsRNA loop” (residues 506–513, Figure S1) in the front, the primer grip (residues 707–712, Figure S1) in the back, and the priming loop (residues 789–805, Figure S1) closing the pocket on the top (front view, Figure 3A,C). This pocket is important for NS5 polymerase *de novo* initiation activity and viral replication.^6,21^ It is also known as motif E or “primer grip” (as it comprises the later), is conserved across RNA viruses and retroviruses, and may be involved in correct positioning of the priming nucleotide ATP during initiation and the primer terminus during elongation.^40–42^ The literature shows that N pocket binders provide good initiation inhibition but slightly poorer inhibition of elongation.^20–23^

**Figure 3:**
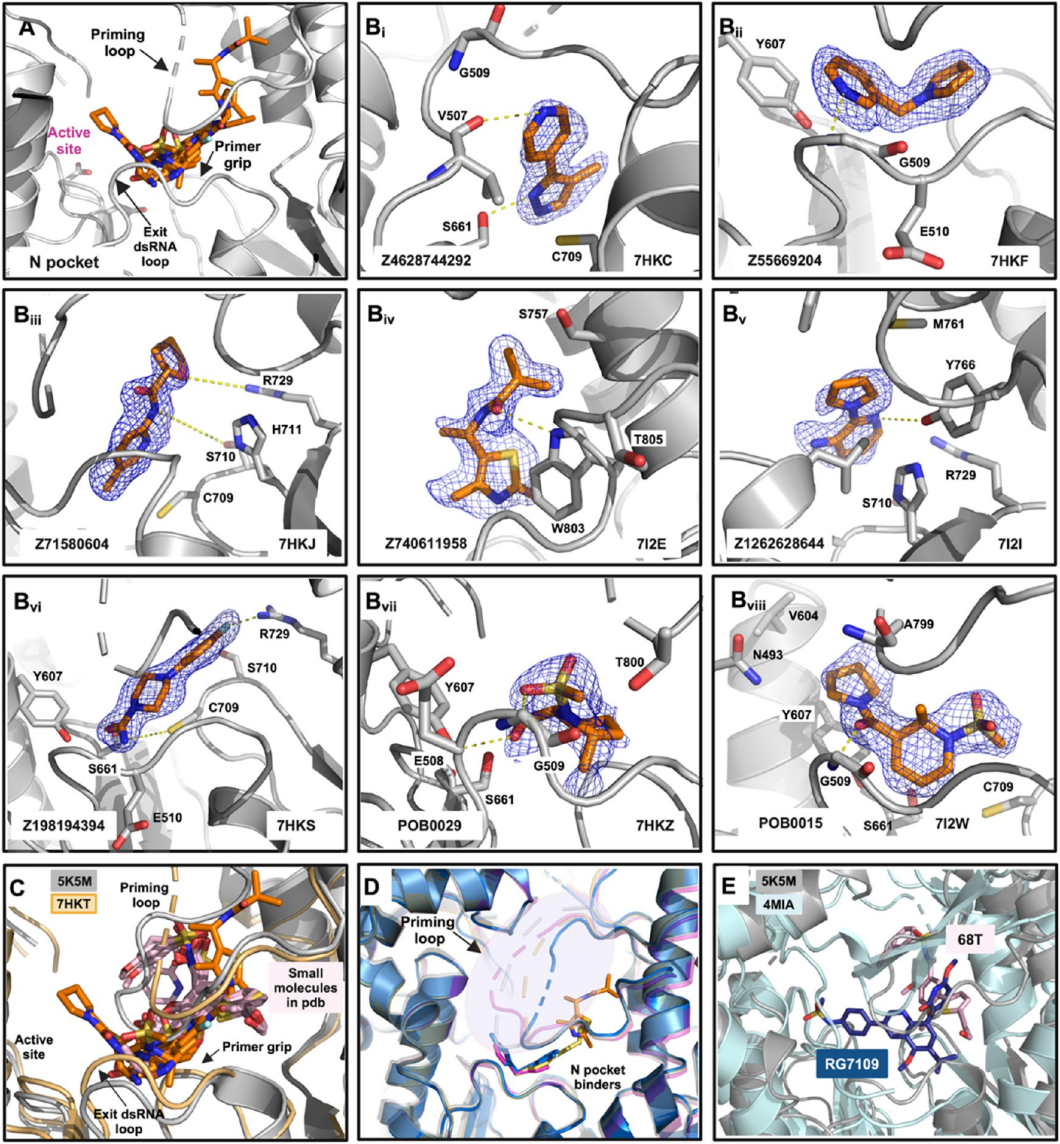
Fragments binding to the N pocket. (A) Superimposition of the N pocket binders and protein elements surrounding them. (B) Each subpanel (B_i_–B_viii_) represents distinct fragments (e.g., Z4628744292, Z55669204, Z71580604, Z740611958, Z1262628644, Z198194394, POB0029, and POB0015) bound in different orientations within the N pocket. The fragments are shown with orange sticks, and the PanDDA event map (at 1σ contour) is shown as a blue mesh. (C) Superimposition of previously reported small molecules from DENV3/2 structures (PDB 5F3T, 5F3Z, 5F41, 5HMW, 5HMX, 5HMY, 5HMZ, 5HN0, 5I3P, 5I3Q, 5K5M, 6H9R, 6H80, and 6IZX) and current fragment screening hits in the same orientation (fragments in orange, small molecules in light pink). Polar interactions are displayed as yellow dashed lines. (D) Ensemble of conformations that the priming loop is adopting in fragment-bound data sets (PDB 7HKF (pink), 7HKJ (yellow), 7I2E (gray cartoon with orange sticks), and 7HKT (blue)). (E) Superposition of DENV2 RdRp N pocket inhibitor 68T (PDB 5K5M) with HCV NS5B RdRp with RG7109 (PDB 4MIA), which is a close analogue of the FDA-approved drug dasabuvir.

We identified a total of nine fragment hits that bind to the N pocket (Figure 3B_i–viii_). The complete list of fragments is provided in Figure S2. As previously commented, the priming loop is disordered in DENV RdRp apo structures, but it is ordered in the presence of N pocket small-molecule binders, as they occupy the N pocket in the interface between the primer grip and the priming loop (Figure 3C). Our fragment hits, on the other hand, mainly form interactions with residues in the area between the exit dsRNA loop and the primer grip and do not display significant interactions with the priming loop. As a result, the priming loop in our liganded structures is partially disordered and shows an ensemble of conformations (Figure 3D). Figure 3C portrays the superimposition of the current fragment hits and the previously reported small molecules in complex with DENV2/3 RdRp.^21–23,43^ We discuss in the “Fragment progression” section the prospects for leveraging all of this chemical matter.

### Fragment Hits in the Conserved RNA Tunnel Site Could Interfere with RNA Template Binding

The RNA tunnel site is a hydrophobic pocket surrounded by helices α5 (preceding motif G, Figure S1) and α8–α9 (immediately after motif F, Figures S1 and 4A), with two flexible loops (motif G or pinky, and the loop before motif B, Figure S1) that move depending on fragment binding (Figure S3A). This binding site, involving the conserved motifs B, G, and F, was previously observed in structures of the DENV3 RdRp with inhibitors NITD107 (PDB 3VWS),^44^ HeE1-2Tyr (PDB 5IQ6),^45^ and NITD-434 (PDB 6XD0).^34^ A total of five binders were identified at this site (Figure 4C,D). The fragment hits mainly display hydrophobic interactions with F399 and F486, and polar interactions, i.e., H-bond with side chains of E494, and with the backbone of Y607 and V604 (Figures 7B_i–v_ and S3B). As this site comprises the pinky finger region or motif G that interacts with the template RNA and plays an important role in restricting the template strand movement, the fragment hits could inhibit or alter the binding and positioning of the RNA template, which is essential for the start of RNA replication, either by direct competition or by alteration of the pinky conformation (Figure 4E). The literature demonstrates that RNA tunnel binders provide good initiation inhibition and slightly poorer inhibition of elongation.^34^

**Figure 4:**
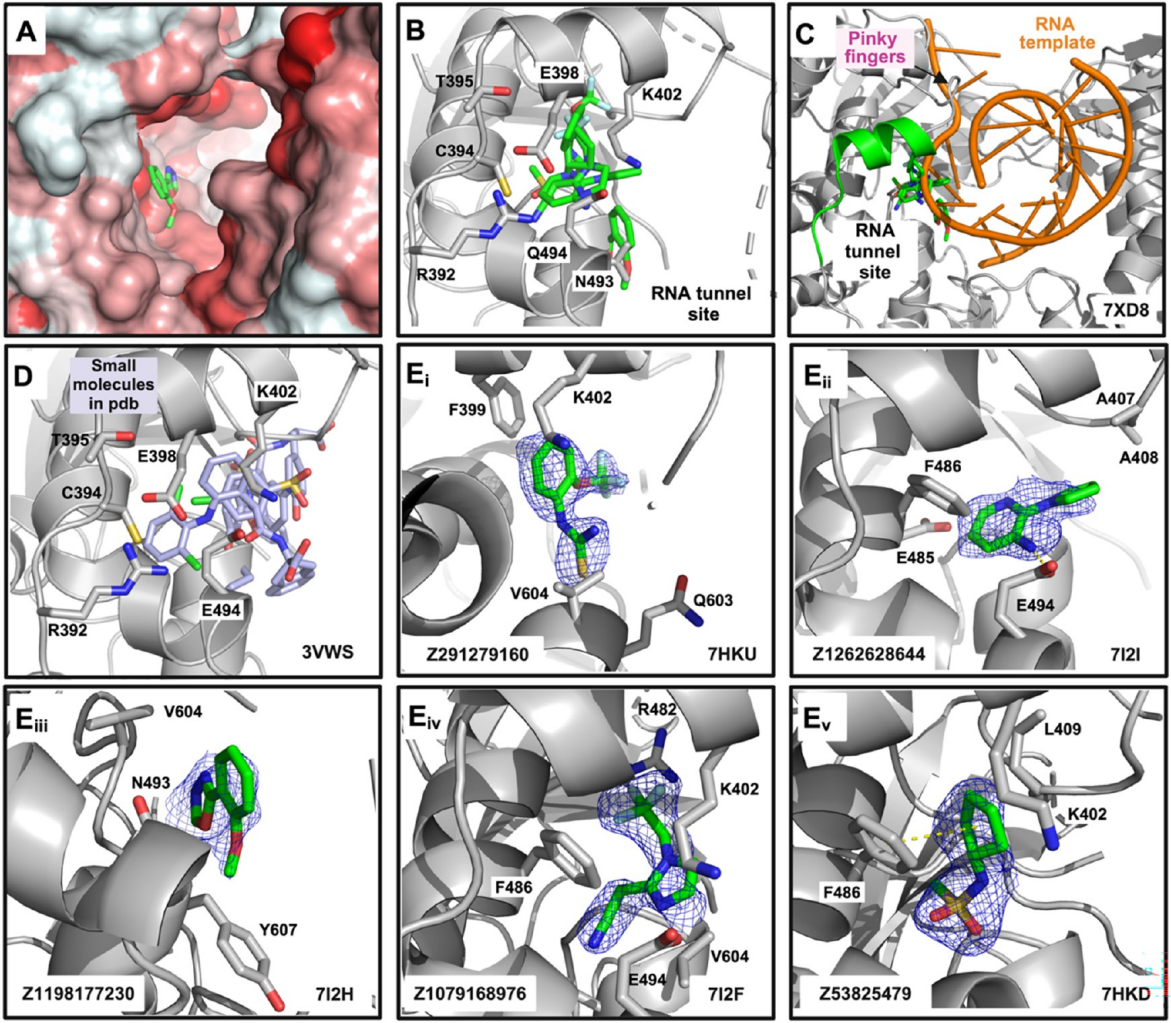
Fragment binding to RNA tunnel site. (A) Surface representation of the RNA tunnel site, colored according to the hydrophobicity scale^46^ (red corresponds to the most hydrophobic atoms, while white corresponds to the less hydrophobic atoms). (B) Superimposition of the RNA tunnel site binders and protein elements surrounding them. (C) Positioning of the RNA tunnel site and its binders near the RNA template (orange) and the pinky fingers. The protein/RNA structure shown corresponds to PDB entry 7XD8. (D) Small molecules from previously solved structures (PDB entries 3VWS and 5IQ6) occupy the same site, underscoring its ligandability. The protein structure shown corresponds to PDB 3VWS. (E) Each subpanel (i–v) represents distinct fragments bound in different orientations within the RNA tunnel site. The fragments are shown as green sticks with the PanDDA event map at 1σ contour as a blue mesh.

### The “Thumb Site II”, a Validated Site in HCV RdRp, Is DENV2 RdRp’s “Hottest” Spot

A total of twenty-nine binders were identified at “thumb site II” (Figures 5A and 1B). Eight representative hits are shown in Figure 5B_i–viii_. The complete list of fragments is provided in Figure S2. Each panel highlights different fragments (yellow sticks) bound to the thumb site II. Nevertheless, a word of caution is needed, as many hits feature at least one interaction that may be mediated by crystal packing, e.g., R698 of the neighboring asymmetric unit (Figure S4A). Along these lines, Figure 5B displays hits that seem to make “genuine interactions” (i.e., independent from crystal packing) with thumb site II. The predominant interactions observed are polar interactions with R770, R856, K887, and R888. Indeed, the thumb site II contains an arginine patch involved in RNA recognition, which, if the interaction is disrupted, results in loss of viral replication, as it occurs for the mutation to alanine of the mentioned R770 and R856.^47^

**Figure 5:**
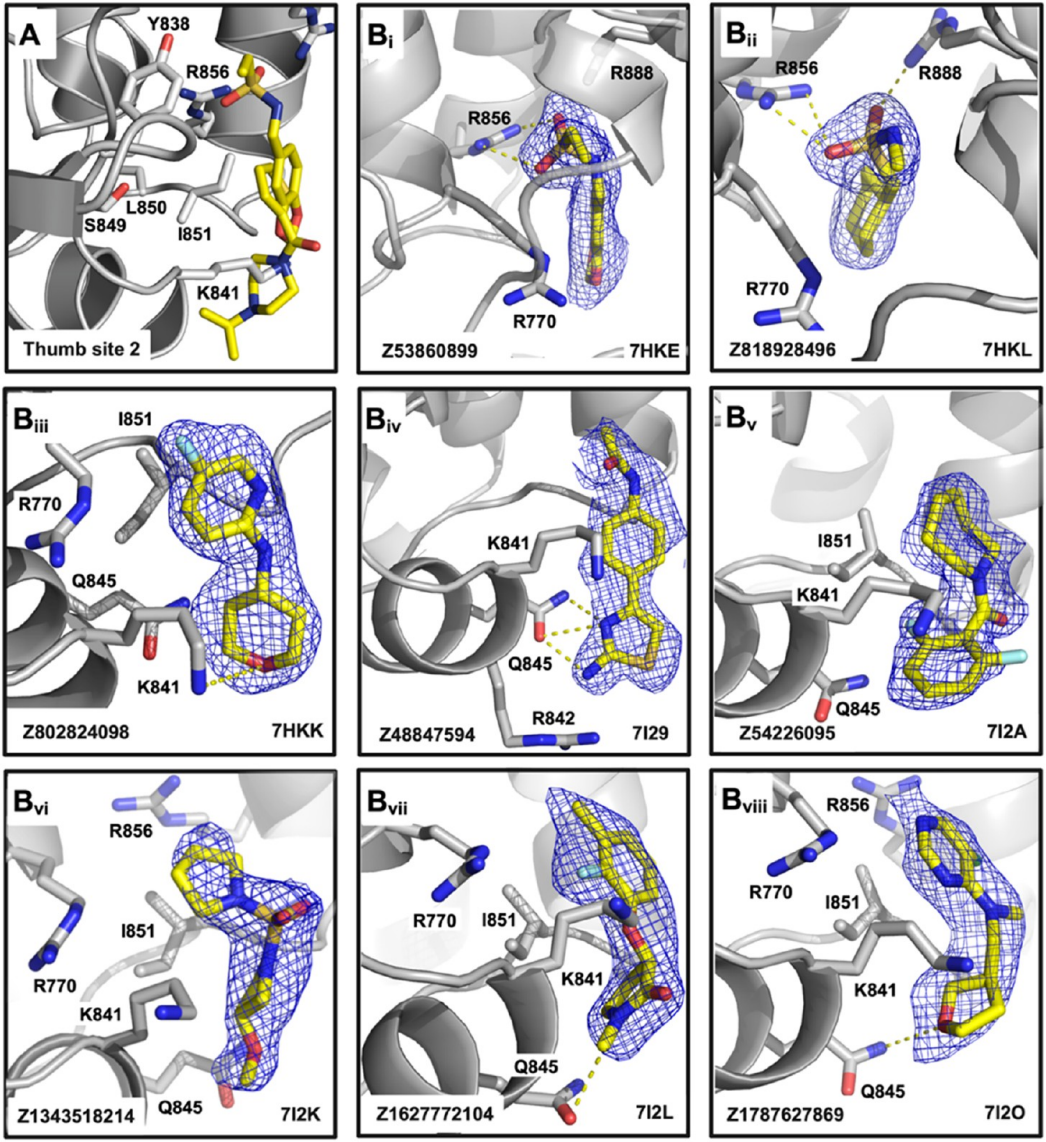
Fragments binding to the thumb site II. (A) Overview of the thumb site II, showing key residues of the protein backbone interacting with bound fragments. (B_i_–B_vii_) Each panel represents a detailed view of individual fragments (yellow sticks) bound to thumb site II, with the corresponding PanDDA event maps at the 1σ contour depicted as blue meshes. Key interactions include hydrogen bonds (yellow dashed lines) with residues such as R770, Q845, K841, and R856, as well as hydrophobic interactions with residues like I851 and L850.

The “thumb site II”, which we named based on the similar location to the analogous pocket in HCV RdRp (Figure 6A), is located on the thumb subdomain near the C-terminus of NS5. This site has been heavily targeted in HCV drug discovery campaigns, with inhibitors such as filibuvir,^48^ lomibuvir,^48^ and radalbuvir^48^ (Figure S4B) having reached phase II clinical trials. The thumb site II seems to prevent structural rearrangements in the HCV RdRp-RNA complex required for the transition from initiation to elongation.^35,36^ Additionally, while their binding to the thumb site II was not acknowledged,^23^ two N pocket binders solved in complex with DENV3 RdRp (PDB 5HMW, 5HMX) also had a second binding site coinciding with the “thumb site II” of DENV2 RdRp (Figure 6B).

**Figure 6:**
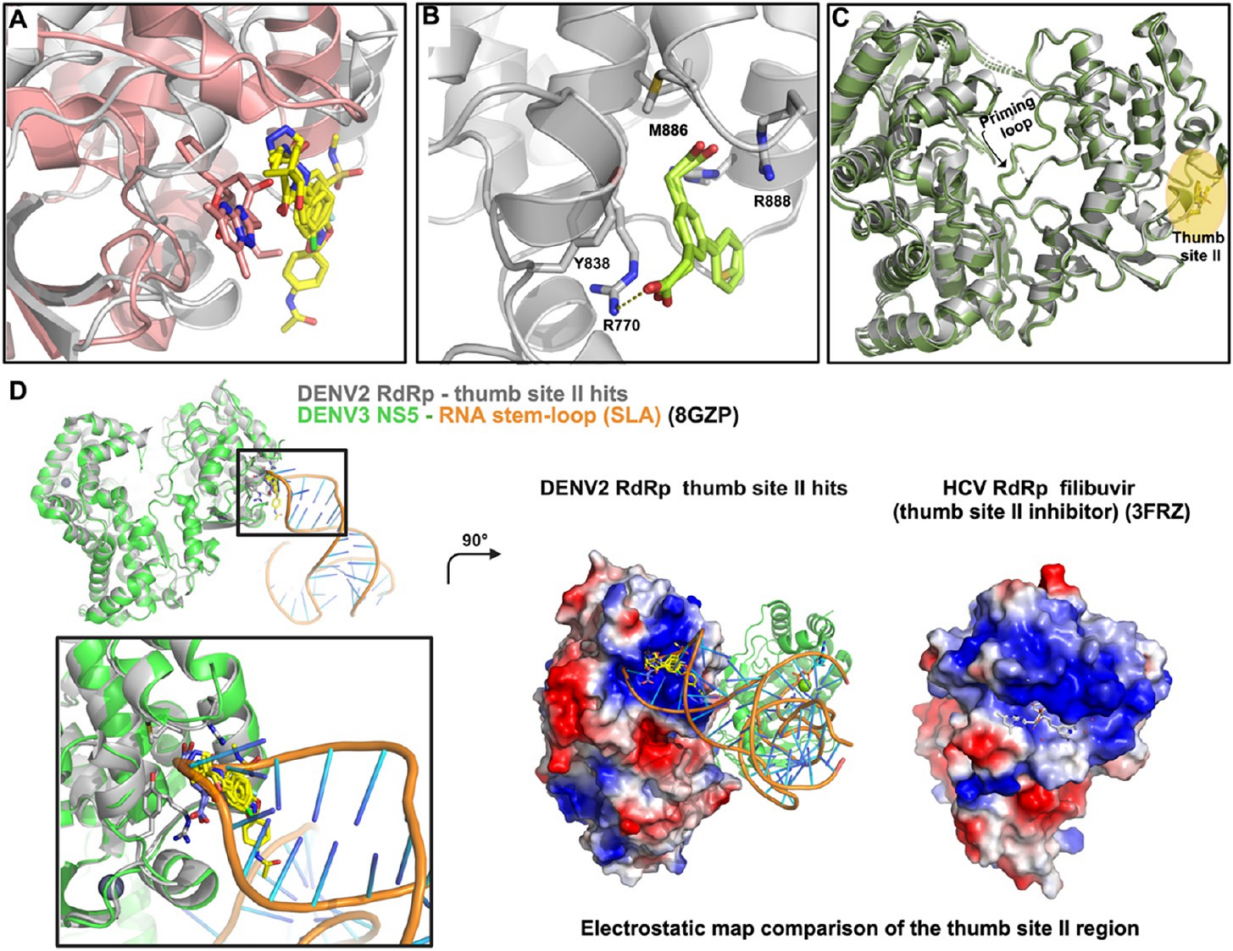
Mechanistic insights into thumb site II hit binding and function. (A) Superposition of the thumb site II hits (yellow sticks) in DENV2 RdRp (gray cartoon) with the HCV NS5B RdRp thumb site inhibitor II inhibitor filibuvir (or PF868554, PDB 3FRZ). Both protein and compound are displayed in light pink. (B) Thumb site II (light green sticks) binding site of the cocrystallized hits with DENV3 RdRp (in gray cartoon and sticks, PDB 5HMW, 5HMX). (C) Comparison of the ground-state (or apo) structure of DENV2 RdRp (gray cartoon, PDB 7I2X) with the DENV2 RdRp (in green cartoon) structures with fragments Z1343518214, Z32327641, and Z32665176 (in yellow sticks, PDB 7I2K, 7I2T, and 7I2U), highlighting the ordered priming loop of the latter. (D) Superposition of the DENV2 RdRp thumb site II hits and the DENV3 NS5 complex with the RNA stem loop (SLA, PDB 8GZP) as indicated in the legend, focusing on the thumb site II binding site. To note that a recent preprint^49^ and deposited structure of the DENV2 NS5/SLA complex (PDB 9DTT) appeared during the preparation of this manuscript. Additionally, the electrostatic potential surfaces of the indicated proteins are displayed to showcase the positively charged nature of both thumb site II binding sites.

### Fragment Progression

The fragment hits found in this campaign show several opportunities for the progression to small-molecule inhibitors of DENV2 RdRp. We have summarized these in Figure 7.

**Figure 7:**
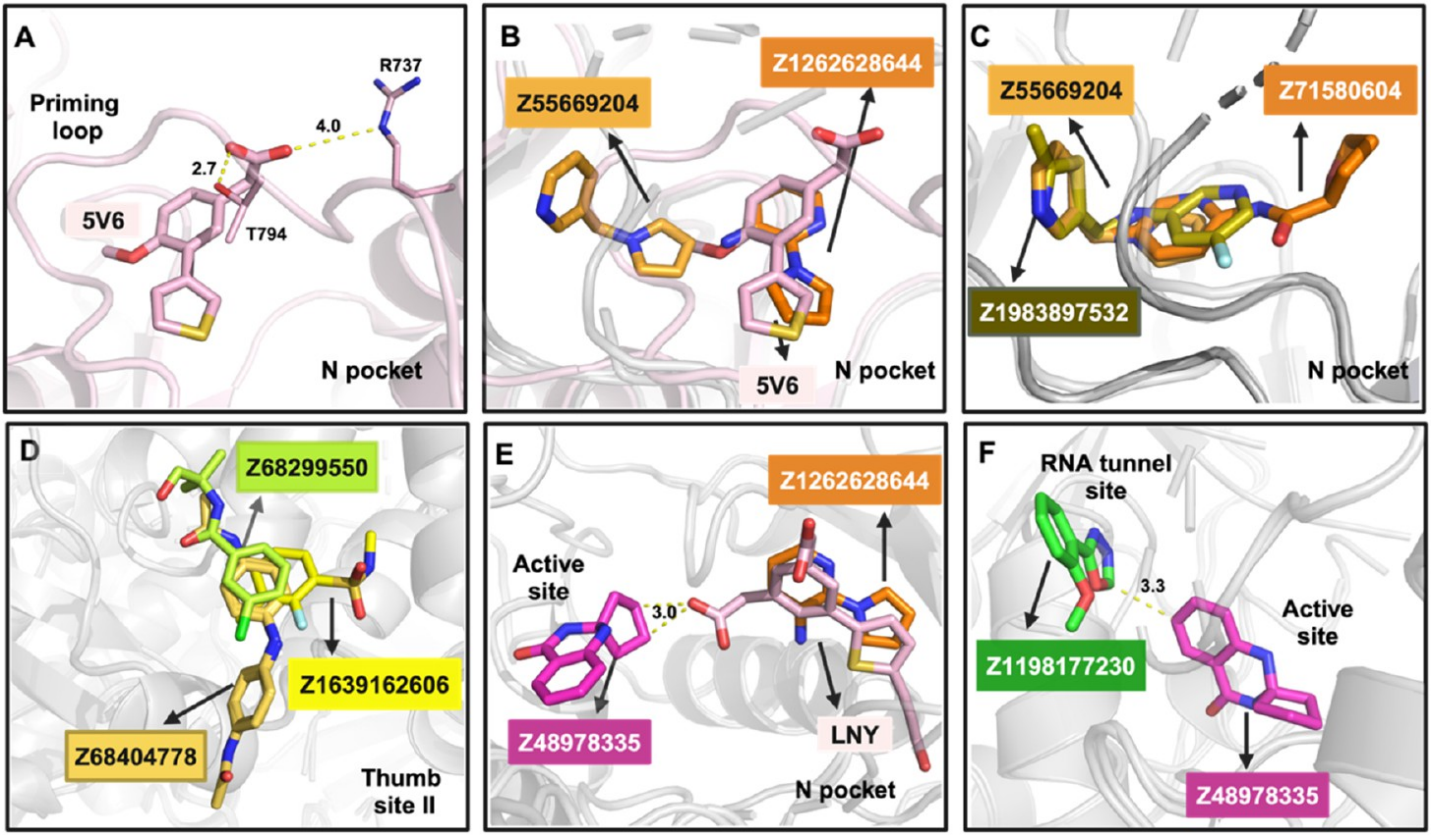
Fragment progression prospects derived from the fragment hits. (A) Acetic acid moiety of the DENV3 RdRp fragment hit 5 V6 (light pink, PDB 5F41) presumably stabilizes the priming loop through polar contacts with residues T794 (main chain) and R737 (side chain). (B, C) N pocket fragment merge prospects originating from the superposition of the indicated DENV2 RdRp-bound structures. (D) Thumb site II fragment merge prospects originating from the superposition of the indicated DENV2 RdRp-bound structures. Fragment linking prospects between the active site hit Z48978335 and (E) indicated N pocket hits or (F) indicated RNA tunnel hit, originating from superposition of the indicated DENV RdRp-bound structures (LNY is a fragment bound to a DENV3 RdRp complex, PDB 5HMY). Minimal distances between fragments to be linked in (E) and (F) are indicated.

Regarding the N pocket, none of our fragment hits stabilize the priming loop (Figure 3A,D). Comparison with the fragment hits found in DENV3 RdRp^22^ shows that Z55669204 has a common core to the biheterocyclic core of the former. However, the DENV3 fragments have an additional acetic acid moiety that presumably stabilizes the priming loop (Figure 7A). Note that further elaboration of this fragment scaffold replaced the acetic acid moiety with an acyl-sulfonamide bioisostere.^21^ Figure 7B shows a fragment merge of the biheterocyclic core of Z1262628644 (with the additional acetic acid moiety) and Z55669204. Such a small molecule would keep the priming loop ordered while extending further toward the exit dsRNA loop. Alternatively, it may be worthwhile to attempt fragment merges with only the current fragment hits, providing a totally novel chemical scaffold (Figure 7C). Regarding the thumb site II, as mentioned, we prioritize the hits with “genuine” interactions (i.e., main interactions are not due to crystal packing) for progression, illustrated with the merge of Z68299550, Z68404778, and Z1639162606 (Figure 7D).

Another appealing avenue is linking fragment hits of the neighboring sites. Concretely, we envision linking the active site hit, Z48978335, with hits of (1) the N pocket and (2) the RNA tunnel site. Regarding (1), we hypothesize that leveraging the second acetic acid moiety of “compound 15” (developed from the aforementioned biheterocyclic core fragment found in the previous DENV3 RdRp Novartis screen^23^) allows one to trace a feasible linking path (Figure 7E). Meanwhile, the active site hit could also be linked to the most proximal hit of the RNA tunnel site, Z1198177230 (Figure 7F).

Finally, the abundance of the hits obtained in these sites will allow to utilize algorithmic design of fragment merges for the three sites, as we and collaborators have done before.^50^ This approach may be especially useful in the case of the RNA tunnel site, with no straightforward manual merges, and to leverage the 29 hits of thumb site II, especially for the hits with a relevant crystal packing interaction component.

## Discussion and Conclusion

Despite the exciting progress initiated by Janssen’s development of JNJ-1802, a first-in-class antiviral agent against dengue,^2^ there is still a lack of therapeutic treatments that effectively combat dengue as well as most other flaviviral diseases.^6^ Therefore, our current work focused on DENV2 RdRp, one of the most conserved targets with potential for broad-spectrum antiviral activity against flaviviruses, which is well aligned with the need for pandemic preparedness collectively.^51,52^

Thus, the 60 hits identified by this crystallographic fragment screen provide novel chemical matter for the development of non-nucleoside small-molecule antivirals that target DENV2 as well as other flaviviral RdRps. Our study has identified four distinct sites, which are presumed to have ligandability, as they have been characterized in related systems (especially HCV RdRp) over the past years (Figure 8). The structural data from our fragment screen are accessible through the Fragalysis web tool (see Data availability section), aligned in a common reference to an analogous Zika virus NS5 RdRp fragment screen and other relevant flaviviral RdRp-ligand structures, allowing their seamless and interactive exploration. These combined efforts may help pave the way for the development of non-nucleoside pan-flaviviral antivirals. We will discuss next some prospects to be pursued.

**Figure 8:**
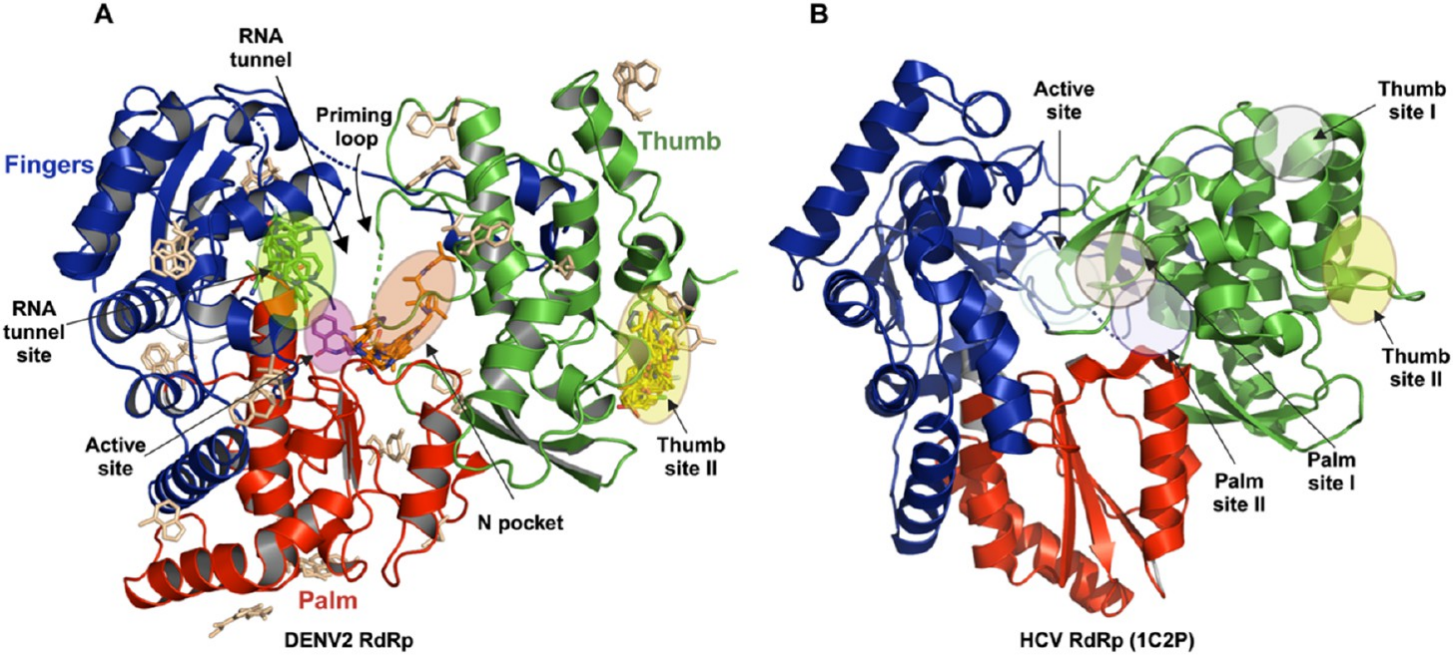
(A) Ribbon representation of the overall structure of DENV2 RdRp (residues 273–891) with identified fragment hits. The subdomains are color coded as follows: fingers in blue, palm in red, and thumb in green. (B) Comparative side-by-side view of the DENV and HCV RdRp structures, highlighting the active site and other allosteric sites.

First, in this fragment screen, we have identified a hit, Z48978335, that binds in the active site of DENV2 RdRp, occupying a spatial region similar to the initiating ATP of the related JEV RdRp^38,39^ and interacting with the GDD-conserved catalytic motif (Figure 2E). This indicates the potential for designing elaborated analogues that can block the initiation step of RNA synthesis. Indeed, flaviviruses have RdRps that initiate RNA synthesis *de novo*, i.e., without the use of a preexisting primer. Given that the active site is highly conserved across all four dengue serotypes and flaviviruses, such hits have the potential to serve as pan-flaviviral inhibitors.

Regarding the previously targeted N pocket in flaviviruses, it is worthwhile to note that the HCV RdRp palm site I, albeit with a different site architecture, has delivered the FDA-approved drug dasabuvir and the related compound RG7109 (Figures 3E and S4B).^53,54^ As mentioned, the retraction of the priming loop from the active site during enzyme elongation may alter the conformation of the N pocket and decrease the affinities of the lead compounds targeting this pocket. Related to this, our fragment hits in the N pocket largely do not engage with the priming loop, in contrast to the previously identified fragments.^21^ Therefore, merging moieties from both our fragment hits and from the previous lead series^22^ may be beneficial to preserve the “best of both worlds”: keeping interaction with the priming loop to “lock it into place” to inhibit initiation but also engaging compounds in the area close to the exit dsRNA loop (Figures 3C and 7A,B). Additionally, the presence of C709 in the primer grip (conserved across all *Flaviviridae* RdRps,^21,22^ Figure S1B) suggests the possibility of designing a novel series of covalent molecules utilizing the Z198194394 scaffold as a starting point, which forms an H-bond with this cysteine (Figure 3B_vi_).

Another attractive vector to explore may be extending the N pocket binders to the active site, merging it with hit Z48978335 (Figure 7E): the engagement with these two regions has the potential to generate tight binding inhibitors. Merging fragments can enhance binding affinity by utilizing multiple interactions between fragments and the protein backbone. Compounds such as Z4628742292, Z55669204, Z198194394, POB0029, and POB0015 (Figure 3B) can serve as connectors, linking the active site and the N pocket.

The RNA tunnel site has also been previously validated as a viable site across different DENV serotypes.^34^ Ligands that bind here appear to interfere with the incoming RNA template and hinder the interaction with the protein backbone during the first steps of RNA polymerization. However, as it is a hydrophobic and relatively enclosed pocket, the fragment and small-molecule hits occupy a similar space (Figure 4). Thus, while there is potential for fragment merging, there is the handicap that they exploit mostly the same limited interactions. As indicated for the N pocket, an intriguing prospect might be the linking of the RNA tunnel site Z1198177230 hit with the aforementioned active site hit (Figure 7F).

Regarding thumb site II, we have found that it is the “hottest spot” with 29 fragment hits bound in addition to two previously published compounds, accounting for many merging opportunities. Nevertheless, as a substantial number have crystal packing mediated interactions, priority should be given to the fragments displaying polar interactions with the arginine patch residues involved in RNA recognition, which have been validated as essential for viral replication.^47^ In this sense, Figure 7D lays out the prospect for a manual merge emerging from these “genuine hits”.

The thumb site II is approximately 35 Å from the active site in both HCV and DENV RdRps, and the mechanism by which binding at this region inhibits polymerase activity was elucidated for HCV through a combination of biochemical and biophysical studies with enzyme constructs with deletions or mutations in the β loop (priming loop notation for HCV) and the C-terminal tail, resulting in a loss of activity in HCV.^35,36^ Indeed, these studies observed that inhibitor binding provides stabilization of an inactive RdRp closed conformation of the β loop propagated via the thumb site II site. In the case of DENV2 RdRp, we have observed how in several of the thumb site II bound structures, the whole priming loop is ordered (contrary to most of the structures of DENV2/3 RdRps, Figure 6C), suggesting an allosteric relationship between this site and the priming loop.

However, our analysis of the recent Dengue RdRp and NS5 structures reveals another potential mechanism. As mentioned, the thumb site II in DENV RdRp has an arginine patch that was observed to be essential for viral replication.^47^ More recently, Osawa and co-workers^15^ have solved cryo-EM structures of NS5 in complex with the flaviviral characteristic 5′-stem-loop structure (SLA). Figure 6D shows how there is a binding interface between a part of the SLA and thumb site II that overlaps with the fragment hits that we have observed in the site. Comparison with the HCV RdRp complex with thumb site II inhibitor filibuvir (as commented, it underwent Phase II clinical trials) shows that in the latter, the pocket is also highly positively charged, which suggests that in HCV (also with a highly structured 5′ UTR region^55^), such a mechanism could also play a role in the mechanism of inhibition.

In summary, our crystallographic fragment screening campaign has comprehensively charted the binding sites of DENV2 RdRp, which have some commonalities and distinct features to the most targeted RNA virus polymerase, the HCV RdRp NS5B (Figure 7). Overall, these findings offer a path for developing pan-flaviviral non-nucleoside RdRps inhibitors, a current unmet need in pandemic preparedness.

## Experimental Section

### Expression and Purification of the Protein

The DENV2 RdRp was codon optimized for *E. coli* expression and synthesized by Genscript in the pET28a vector. The pET28a-DENV2 RdRp construct containing the gene of interest was transformed into *E. coli* BL21 (DE3) star cells and plated on LB agar with 50 μg/mL of kanamycin. A single colony was inoculated in 10 mL of Luria Broth media for primary culture, followed by overnight incubation at 37 °C. This primary culture (*A*_600_ = ∼1) was used to inoculate 1 L of the LB media (secondary culture). The secondary culture was incubated at 37 °C at 180 rpm of shaking until its optical density at 600 nm reached a value of 0.6. The secondary culture was induced with 0.4 mM IPTG (Bio) and incubated at 16 °C for 16 h at 180 rpm of shaking.

The cells were harvested at 4000*g* and resuspended in a Lysis buffer of 20 mM HEPES, pH 7.0, 300 mM NaCl, 10% (v/v) glycerol, and EDTA-free protease inhibitor cocktail. IGEPAL CA-630 was then added to a final concentration of 0.1% (v/v), followed by the addition of 0.05% (v/v) polyethylenimine (PEI) to precipitate nucleic acid. The lysate was slowly stirred at 4 °C for 15 min, sonicated, and then centrifuged for 60 min at 44,000*g* (4 °C). The supernatant was purified by nickel-nitrilotriacetic acid (Ni-NTA) affinity chromatography by washing the unbound protein with the buffer supplemented with 10 mM imidazole. The RdRp was eluted in a stepwise gradient manner ranging from 40 to 200 mM imidazole. For removing the N-terminal His tag, in-house-produced HRV14 3C protease was added to the pooled fractions containing the RdRp in a 1:50 ratio; the mixture was dialyzed overnight against the buffer, 20 mM HEPES at pH 7.0, 300 mM NaCl, and 1 mM tris­(2-carboxyethyl) phosphine (TCEP). The uncut protein pool was separated from the cleaved protein via reverse Ni-NTA. The cleaved RdRp protein was further purified by size exclusion chromatography using 20 mM HEPES, pH 7.0, 300 mM NaCl, and 5 mM TCEP. SDS-PAGE analysis of the resulting RdRp indicated that the protein was purified to homogeneity.

### Crystallization and Structure Determination

Crystals were grown in a crystallization solution comprising 0.35 M MgCl_2_ along with 0.1 M MES at pH 6.6, and 10% PEG 4000 maintained at 4 °C for 1 week. An initial model was obtained at a resolution of 1.6 Å (collected at APS beamline 23ID-D), processed within the *I*222 space group using HKL 2000,^56^ and subsequently solved through molecular replacement employing the PDB 5K5M as a reference model with Phaser^57^ and refined with phenix.refine.^58^

For fragment screening, seeds were prepared from crystals that were aspirated and vortexed with 100 μL of the crystallization solution, along with glass beads. After optimization, crystals grew reproducibly at 20 °C using sitting-drop vapor diffusion in MRC 3 Lens Crystallization plates (SWISSCI) with 300 nL of the aforementioned reservoir solution, with a 1:2 v/v ratio of protein to crystallization solution. This process led to the acquisition of viable crystals in approximately 80% of the drops within 48 h of the plate setup. To determine solvent tolerability, crystals were incubated with DMSO ranging from 10 to 20% for periods of 1–3 h at room temperature.

### Fragment Screening

The crystallographic fragment screening was performed using the XChem platform available at Diamond Light Source.^24,59^ Crystal soaking was done by transferring fragments from the respective library plate to the crystal drop using ECHO Liquid Handler^60^ at a proportion of 10% v/v DMSO. The fragment libraries chosen were selected to maximize the screening of the chemical space, with varied design criteria, aiming at higher hit rates and a more efficient lead optimization. DSi-Poised^61^ is a poised fragment library that enables rapid synthetic expansion. Minifrags^62^ are ultrawater-soluble, chemically diverse, and ultralow-molecular-weight fragments. Fraglites^63^ are a set of halogenated compounds expressing paired hydrogen-bonding motifs. The Peplites^64^ library was developed to map noncovalent interactions of amino acid side chains in protein–protein interaction hotspots. York3 ^65^ contains pyrrolidine and piperidine fragments that occupy under-represented areas of fragment space. SpotXplorer^66^ presents a minimal diverse set of commercial fragments covering the majority of the experimental binding pharmacophores to be used for the identification of fragment starting points for drug discovery targets. Finally, Halo^67^ contains halogenated fragments (including the “universal fragment” 4-bromopyrazole) that have been shown to bind to two or more targets experimentally, leveraging the unusually high hit rates of halogenated fragments in fragment screening campaigns.

Crystals were incubated for 1–3 h at room temperature and harvested using the Crystal Shifter^68^ (Oxford Lab Technologies), mounted on loops, and cryo-cooled in liquid nitrogen. All data was collected at I04–1 beamline at Diamond Light Source at 100 K and processed with automated pipelines. All further analysis was performed using XChemExplorer.^69^ Initial density maps were generated using DIMPLE^70^ and with PDB 5K5M (with heteroatoms stripped) as template model. Ligand restraints were generated with ACEDRG^71^ and GRADE.^72^ A ground-state model was constructed utilizing PanDDA by amalgamating 100 apo data sets. This model, deposited under the code 7I2X (Table S1), served as the template for molecular replacement for the full analysis. Event maps were calculated with PanDDA,^26,73^ and ligands were modeled using Coot.^74^ Models were refined with Refmac^75^ using ensemble refinement^75^ (in line with our established protocols for modeling conformational heterogeneity^76^) and phenix.refine,^58^ and models and quality annotations cross-reviewed. To note that due to current limitations in the PDB deposition system, only the bound state was deposited together with the experimental structure factors and the final refined mtz file (the PanDDA event map is stored as distinct data blocks in one cif file). As a result, the validation metrics derived from wwpdb may not fully reflect the quality of the ensemble refinement and could be misleading, particularly metrics such as R-factors, RSRZ, and correlation coefficients. Thus, we have made PanDDA event maps available at 10.5281/zenodo.16055906. Coordinates and structure factors for all data sets are deposited in the Protein Data Bank (PDB, Group deposition IDs G_1002317 and G_1002341). Data collection and refinement statistics, PBD codes, and ligand identifiers are available in Table S1. Figures were prepared with PyMOL (The PyMOL Molecular Graphics System, Version 2.5 Schrödinger, LLC, New York, NY, USA) and BioRender (created with BioRender.com, Toronto, ON, Canada).

## Supplementary Material







## Data Availability

The coordinates and structure factors have been deposited in the Protein Data Bank, under group deposition IDs G_1002317 and G_1002341. The accession codes and other descriptors are listed in Tables S1 and S2, respectively. Additionally, the structures have been made available on Fragalysis: https://fragalysis.diamond.ac.uk/viewer/react/preview/target/DENV2_NS5_RdRp/tas/lb32633-1 and https://fragalysis.diamond.ac.uk/viewer/react/preview/target/Flavi_NS5_RdRp/tas/lb32633-1. Additionally, we provide a Zenodo directory containing all the bound-state model pdb files and associated mtz files, all the event maps, and all ligand restraint files for all the deposited data sets at 10.5281/zenodo.16055906.

## Notes

Figures S1–S4 (PDF)
Tables S1 and S2 (XLSX)
Additional data (CSV)

## ∇

Department of Biochemistry, Vanderbilt University School of Medicine, Nashville, Tennessee 37232, United States

## Abbreviations Used

ASU: asymmetric unit
DMSO: dimethyl sulfoxide
DENV: dengue viruses
ER: endoplasmic reticulum
HCV: hepatitis C virus
MTase: methyltransferase
Ni-NTA: nickel-nitrilotriacetic acid
NS5: nonstructural protein 5
NS: nonstructural
PEI: polyethylenimine
PanDDA: Pan-Data set Density Analysis
PDB: Protein Data Bank
RdRp: RNA-dependent RNA polymerase
STAT2: signal transducer and activator of transcription 2
SLA: stem-loop structure
TCEP: tris­(2-carboxyethyl) phosphine
UTR: untranslated regions
HEPES: 2-[4-(2-hydroxyethyl)­piperazin-1-yl]­ethanesulfonic acid
