# Supplementary material for: Crystallographic Fragment Screening of the Dengue Virus Polymerase Reveals Multiple Binding Sites for the Development of Non-nucleoside Antiflavivirals
Source: J Med Chem. 2025 Sep 2;68(17):18356–69. doi: 10.1021/acs.jmedchem.5c01014 (PMC12434669; doi:10.1021/acs.jmedchem.5c01014)
Supplement: Supplementary file 1 [file jm5c01014_si_001.pdf]

Supplementary Information

Crystallographic fragment screening of the dengue virus polymerase reveals multiple binding sites for the development of non-nucleoside antiflavivirals

Manisha Saini[1,2,*], Jasmin C. Aschenbrenner[3,4*], Francesc Xavier Ruiz[1,2,*,†], Ashima Chopra[1,2,‡], Anu V. Chandran[3,4], Peter G. Marples[3,4], Blake H. Balcomb[3,4], Daren Fearon[3,4,†], Frank von Delft[3,4,5,†], Eddy Arnold[1,2,†]

[1]Center for Advanced Biotechnology and Medicine, Rutgers, the State University of New Jersey, Piscataway, NJ, 08854, USA

[2]Department of Chemistry and Chemical Biology, Rutgers, the State University of New Jersey, Piscataway, NJ, 08854, USA

[3]Diamond Light Source, Harwell Science and Innovation Campus, Fermi Ave, Didcot OX11 0DE, UK

[4]Research Complex at Harwell, Harwell Science and Innovation Campus, Fermi Ave, Didcot, OX11 0FA, UK

[5]Centre for Medicines Discovery, Nuffield Department of Medicine Research Building, Old Road Campus, Headington, Oxford, OX3 7FZ, UK

[*]These authors contributed equally to this work

[‡]Present address: Department of Biochemistry, Vanderbilt University School of Medicine, Nashville, TN, 37232, USA

[†]Corresponding authors. Email: arnold@cabm.rutgers.edu (E.A.); frank.von-delft@diamond.ac.uk (F.vD.); daren.fearon@diamond.ac.uk (D.F.); xavier@cabm.rutgers.edu (F.X.R.)

This PDF file includes:

Supplementary Figure 1 to 4

A

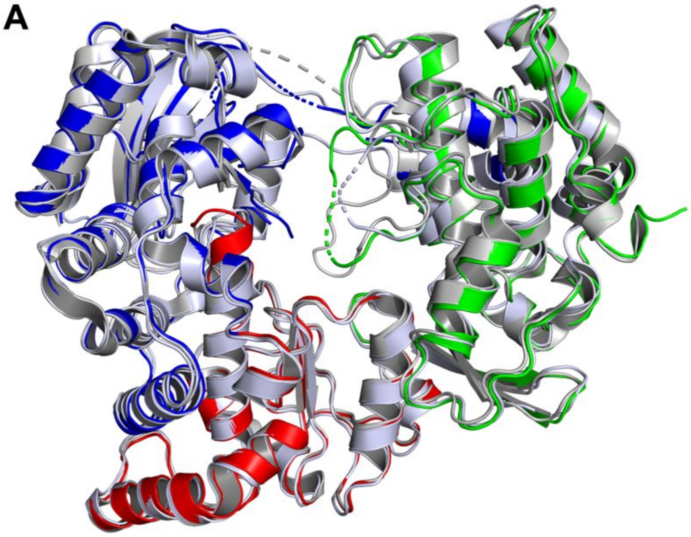

A.1

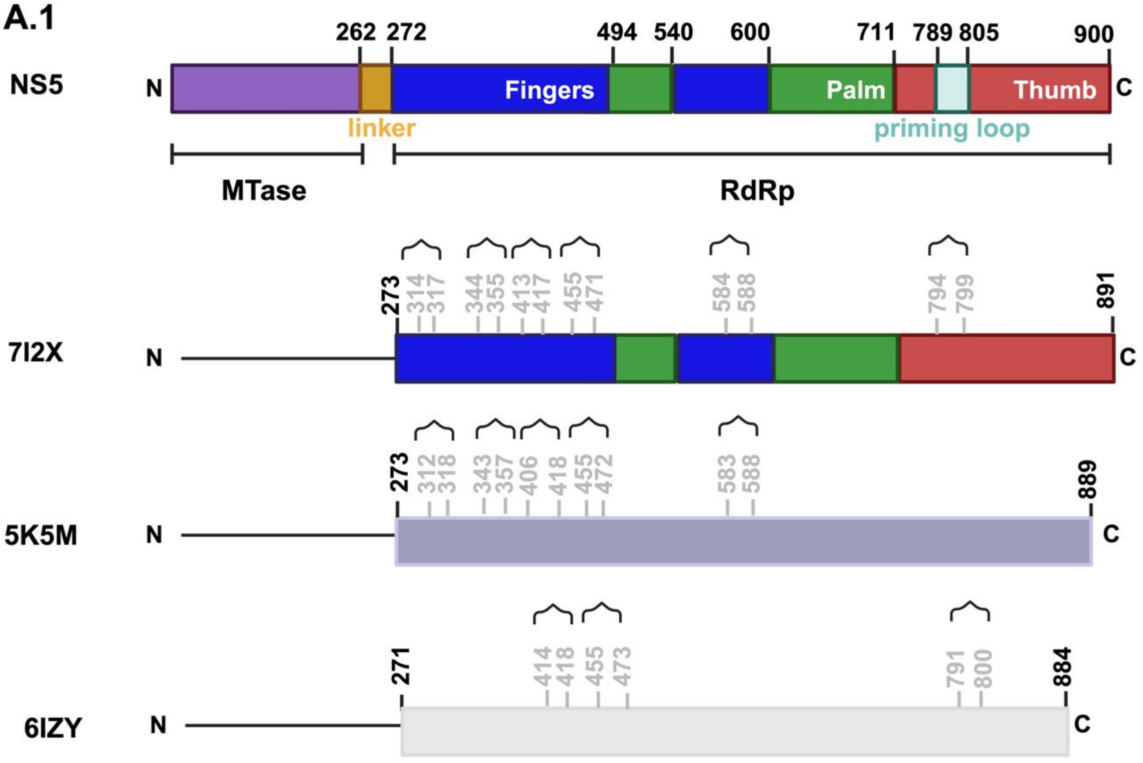
262
272
494
540
600
711
789
805
900
NS5
N
Fingers
Palm
Thumb
C
linker
priming loop
MTase
RdRp
7I2X
N
273
314
317
344
355
413
417
455
471
584
588
794
799
891
C
5K5M
N
273
312
318
343
357
406
418
455
472
583
588
889
C
6IZY
N
271
414
418
455
473
791
800
884
C

B

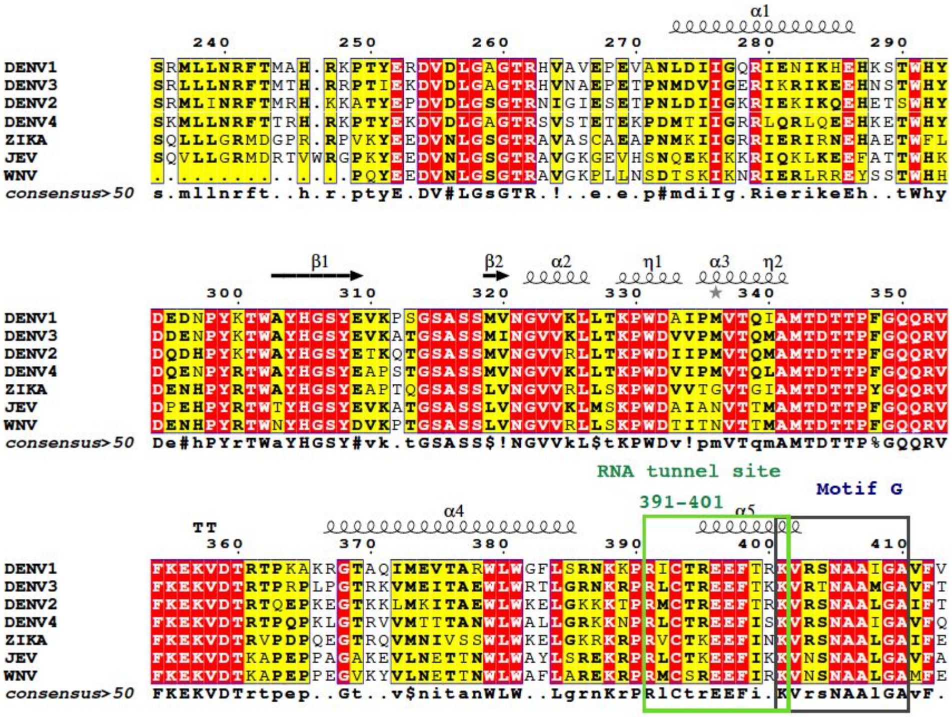

α1
240
250
260
270
280
290
DENV1 SRMLLNRFTMAH.RKPTYERDVDLGAGTRHVAVEPEVANLDIIGQRIENIKHEHKSTWHY
DENV3 SRLLLNRFTMTH.RRPTIEKDVDLGAGTRHVNAEPETPNMDVIGERIKRIKEEHNSTWHY
DENV2 SRMLINRFTMRH.KKATYEPDVDLGSGTRNIGIESETPNLDIIGKRIEKIKQEHETSWHY
DENV4 SKMLLNRFTTRH.RKPTYEKDVDLGAGTRSVSTETEKPDMTIIGRRLQRLQEEHKETWHY
ZIKA SQLLLGRMDGPR.RPVKYEEDVNLGSGTRAVASCAEAPNMKIIGRRIERIRNEHAETWFL
JEV SQVLLGRMDRTVWRGPKYEEDVNLGSGTRAVGKGEVHSNQEKIKKRIQKLKEEFATTWHK
WNV .............PQYEEDVNLGSGTRAVGKPLLNSDTSKIKNRIERLRREYSSTWHH
consensus>50 s.mllnrft..h.r.ptyE.DV#LGsGTR.!..e.e.p#mdiIg.RierikeEh..tWhy
β1
β2
α2
η1
α3
η2
300
310
320
330
340
350
DENV1 DEDNPYKTWAYHGSYEVKPSGSASSMVNGVVKLLTKPWDAIPMVTQIAMTDTTPFGQQRV
DENV3 DDENPYKTWAYHGSYEVKATGSASSMINGVVKLLTKPWDVVPMVTQMAMTDTTPFGQQRV
DENV2 DQDHPYKTWAYHGSYETKQTGSASSMVNGVVRLLTKPWDIIPMVTQMAMTDTTPFGQQRV
DENV4 DQENPYRTWAYHGSYEAPSTGSASSMVNGVVKLLTKPWDVIPMVTQLAMTDTTPFGQQRV
ZIKA DENHPYRTWAYHGSYEAPTQGSASSLVNGVVRLLSKPWDVVTGVTGIAMTDTTPYGQQRV
JEV DPEHPYRTWTYHGSYEVKATGSASSLVNGVVKLMSKPWDAIANVTTMAMTDTTPFGQQRV
WNV DENHPYRTWNYHGSYDVKPTGSASSLVNGVVRLLSKPWDTITNVTTMAMTDTTPFGQQRV
consensus>50 De#hPYrTWaYHGSY#vk.tGSASS$!NGVVkL$tKPWDv!pmVTqmAMTDTTP%GQQRV
RNA tunnel site
391-401
Motif G
TT
α4
α5
360
370
380
390
400
410
DENV1 FKEKVDTRTPKAKRGTAQIMEVTARWLWGFLSRNKKPRICTREEFTRKVRSNAAIGAVFV
DENV3 FKEKVDTRTPRPLPGTRKVMEITAEWLWRTLGRNKRPRLCTREEFTKKVRTNAAMGAVFT
DENV2 FKEKVDTRTQEPKEGTKKLMKITAEWLWKELGKKKTPRMCTREEFTRKVRSNAALGAIFT
DENV4 FKEKVDTRTPQPKLGTRVVMTTTANWLWALLGRKKNPRLCTREEFISKVRSNAAIGAVFQ
ZIKA FKEKVDTRVPDPQEGTRQVMNIVSSWLWKELGKRKRPRVCTKEEFINKVRSNAALGAIFE
JEV FKEKVDTKAPEPPAGAKEVLNETTNWLWAYLSREKRPRLCTKEEFIKKVNSNAALGAVFA
WNV FKEKVDTKAPEPPEGVKYVLNETTNWLWAFLAREKRPRMCSREEFIRKVNSNAALGAMFE
consensus>50 FKEKVDTrtpep..Gt..v$nitanWLW..LgrnKrPRlCtrEEFi.KVrsNAAlGAvF.

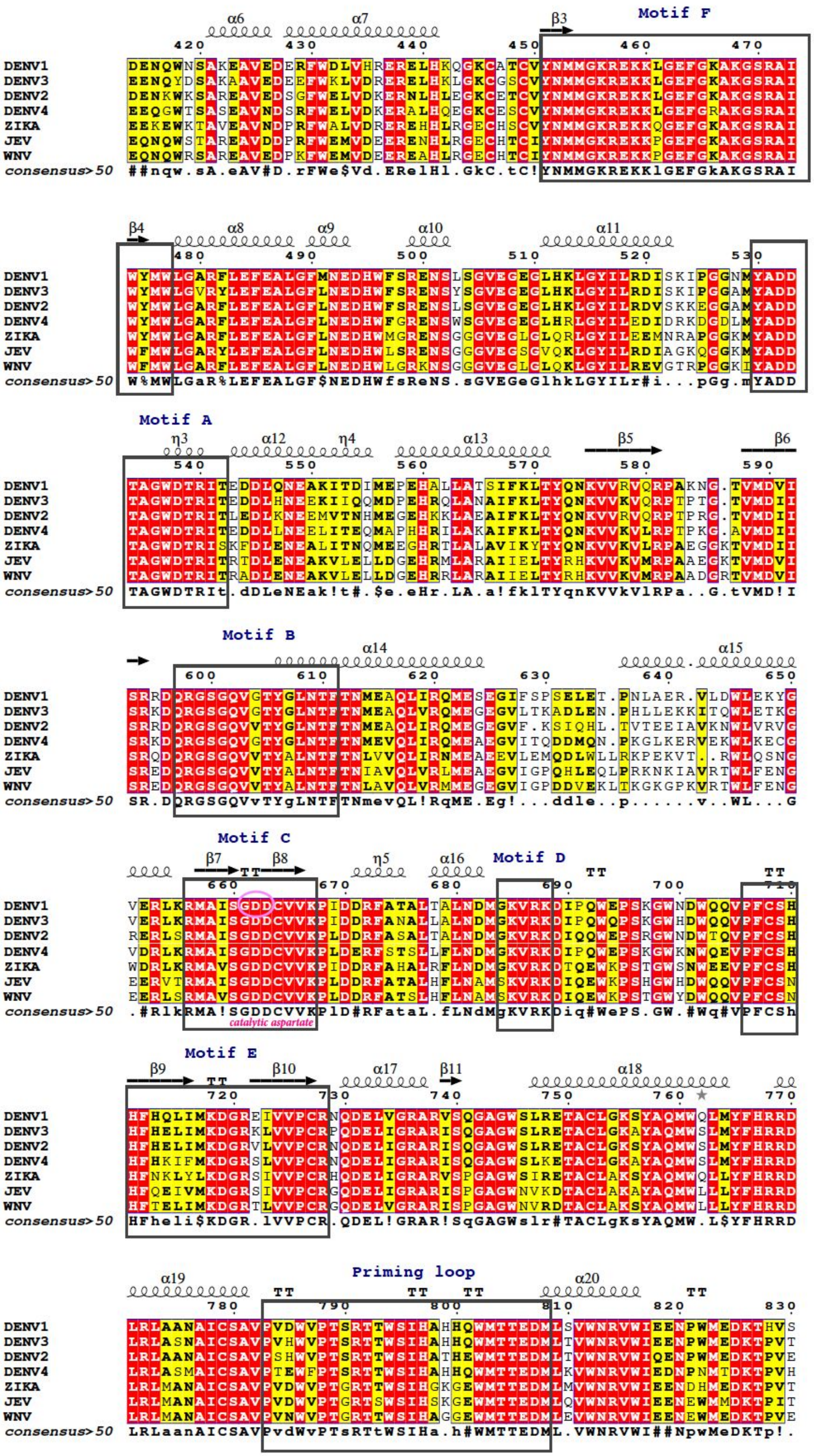

Motif F
Motif A
Motif B
Motif C
Motif D
Motif E
Priming loop
catalytic aspartate
DENV1
DENV3
DENV2
DENV4
ZIKA
JEV
WNV
consensus>50

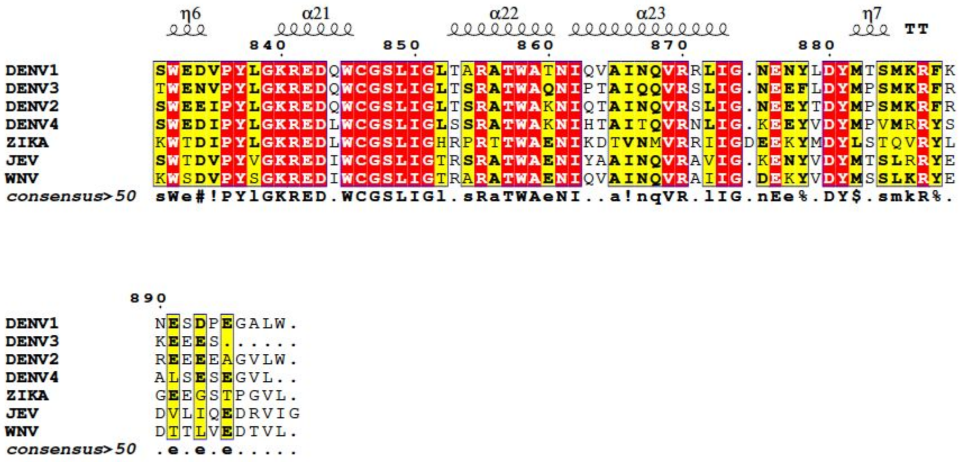

Figure S1. Structural comparison of the unbound three-dimensional structure of DENV2 RdRp (PDB: 7I2X) with previously reported structures (PDB: 5K5M and 6IZY). A) The overlay highlights structural similarities and differences, providing insights into conformational variations among these RdRp structures. A.1) Non-observed regions are highlighted in gray. B) Multiple sequence alignment of DENV2 with all its serotypes and close homologs. The conserved structural motifs of RdRps, i.e., A–G, are highlighted in box. The figure was generated using ESPript 3 (https://espript.ibcp.fr/).

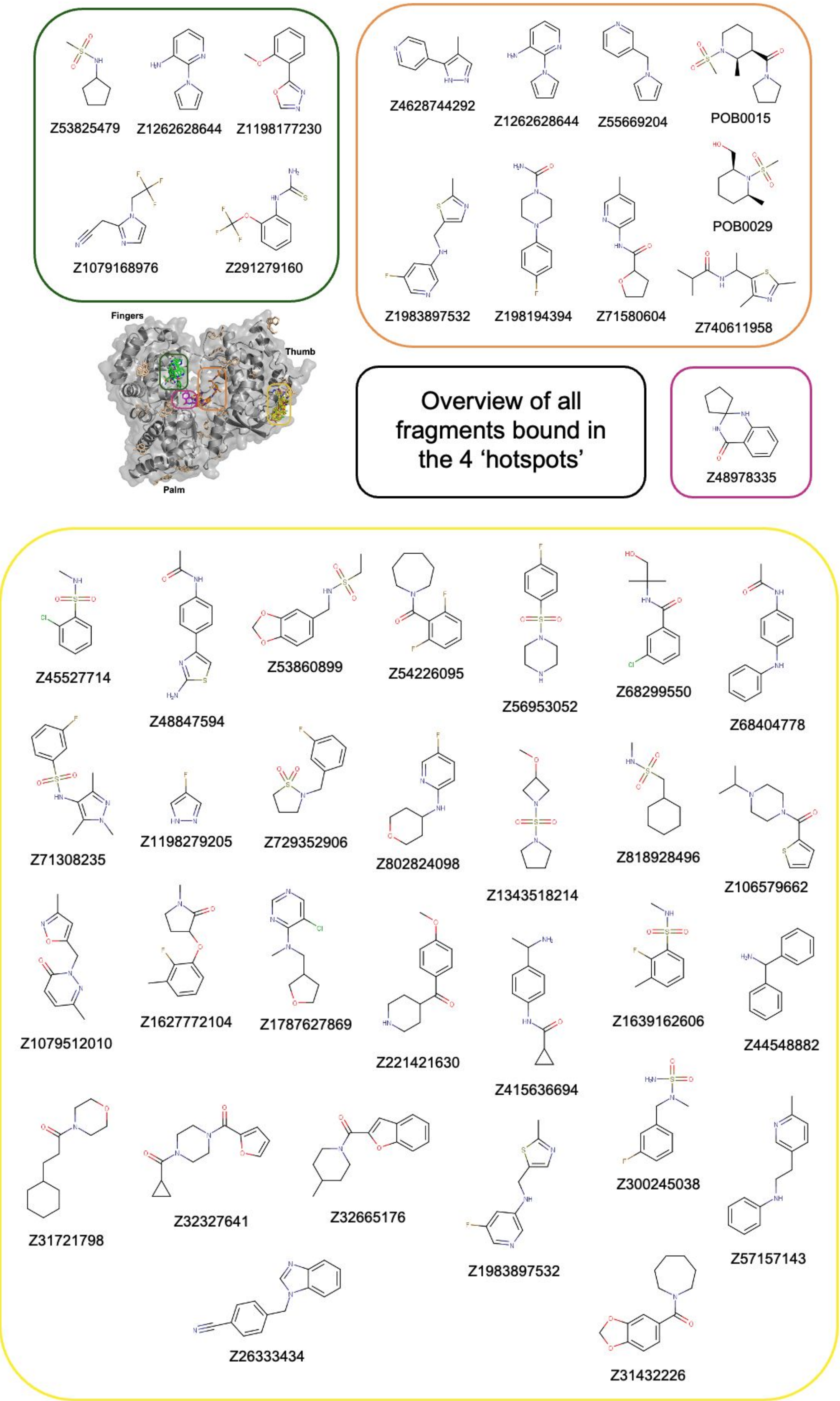

Figure S2. Full list of the fragment hits of the four nominated sites. Pink box: Active site; orange box: N pocket or Primer grip site; green box: RNA tunnel site; yellow box: Thumb site II.

A

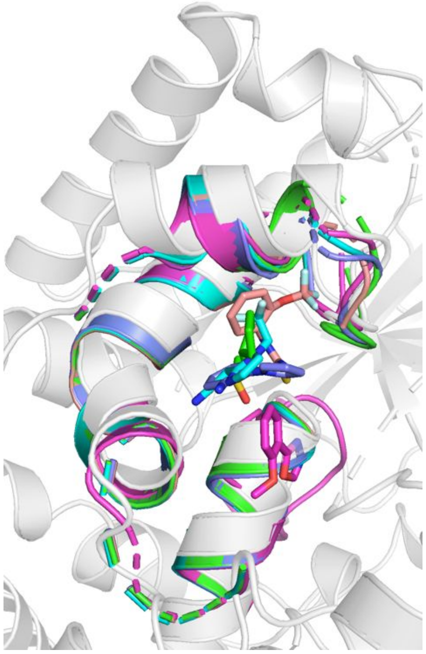

B

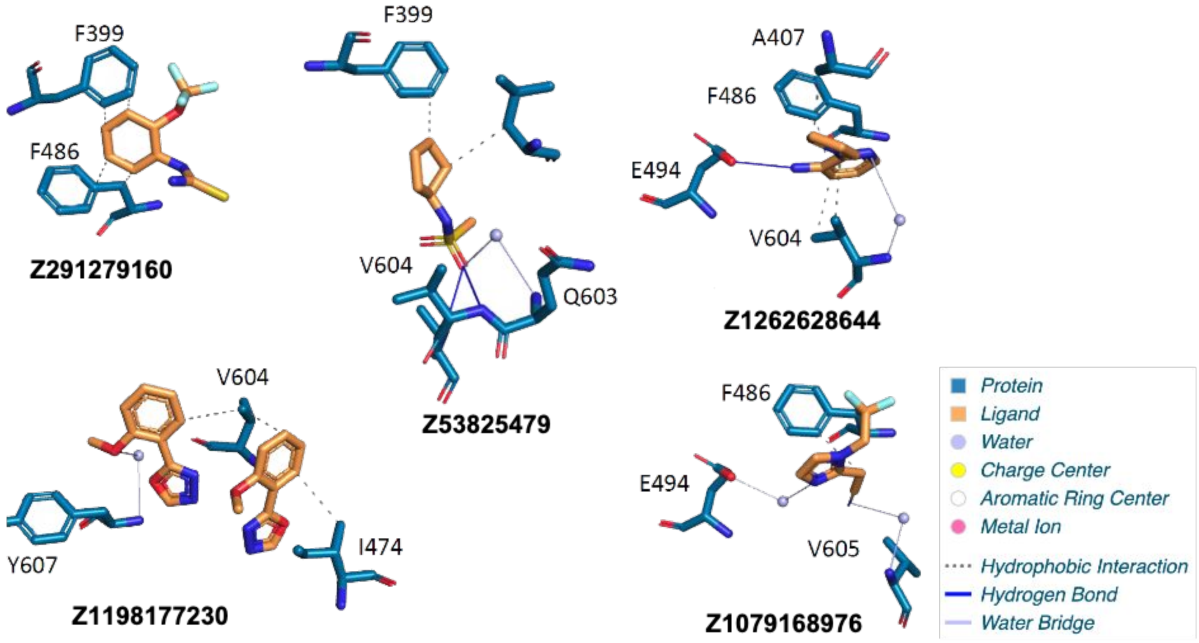

Figure S3. DENV2 RdRp RNA tunnel site detailed view. A) Conformational variation of the RNA tunnel site upon fragment binding. Legend: Z53825479 - PDB 7HKD (green), Z1079168976 -PDB 7I2F (cyan), Z1198177230 - PDB 7I2H (pink), Z1262628644 - PDB 7I2I (violet), Z291279160 - PDB 7HKU (light pink). B) Detailed interactions for each fragment hit in the RNA tunnel site, using the PLIP server (https://plip-tool.biotec.tu-dresden.de/plip-web/plip/index).

A

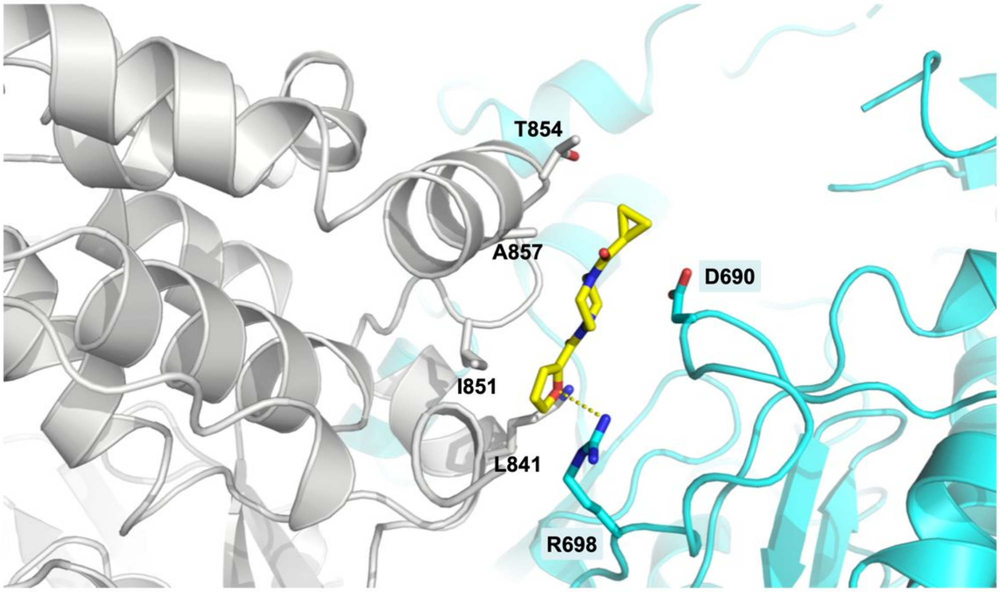

B

**Compound name (and binding site)**	**Structure**	**Pubchem ID**
**Dasabuvir** **(Primer grip)**	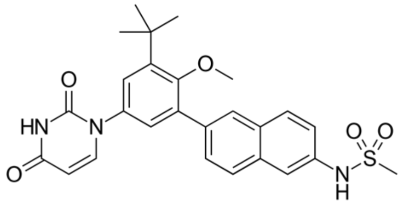	56640146
**RG7109** **(Primer grip)**	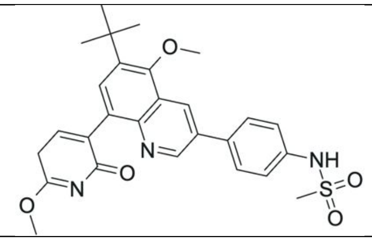	137348037
**Filibuvir** **(Thumb site II)**	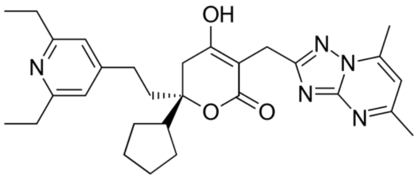	54708673

**Lomibuvir**<br><br>**(Thumb site II)**	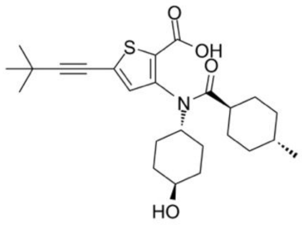	24798764
**Radalbuvir**<br><br>**(Thumb site II)**	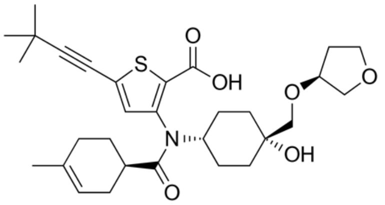	53259022

Figure S4. A) Certain binding interactions at the DENV2 RdRp thumb site II were influenced by crystal packing such as with R698 of the neighboring asymmetric unit (cyan). B) Known HCV non-nucleoside inhibitors (NNIs) with their two-dimensional structures and pubchem IDs.
